# PEGylated Chrysin: In Vitro Biological Activity and Mechanistic Insights in HepG2 Cells

**DOI:** 10.1002/jbt.71169

**Published:** 2026-11-04

**Authors:** Fátima Belén Gasser, Julia Oggero, María Sol Renna, María Florencia Beltramino, Candela Simonetto, Lucía Dominguez, Hugo Héctor Ortega, Santiago Eduardo Vaillard, Victoria Anahí Vaillard, María Eugenia Baravalle

**Affiliations:** ^1^ Centro de Medicina Comparada, Instituto de Ciencias Veterinarias del Litoral (ICIVET) Universidad Nacional del Litoral and Consejo Nacional de Investigaciones Científicas y Técnicas (CONICET). Esperanza Santa Fe Argentina; ^2^ Instituto de Desarrollo Tecnológico para la Industria Química (INTEC) Universidad Nacional del Litoral and Consejo Nacional de Investigaciones Científicas y Técnicas (CONICET) Santa Fe Argentina; ^3^ Laboratorio de Microscopia Aplicada a Estudios Moleculares y Celulares (LAMAE) Instituto de Investigación y Desarrollo en Bioingeniería y Bioinformática (IBB), UNER. Oro Verde Entre Ríos Argentina; ^4^ Centro Universitario Gálvez Universidad Nacional del Litoral (UNL), Gálvez Santa Fe Argentina

**Keywords:** cytotoxic activity, flavonoids, hepatocellular carcinoma, membrane cell damage, PEGylated chrysin

## Abstract

PEGylated chrysin, a natural flavonoid conjugated with methoxypolyethylene glycol, exhibits in vitro cytotoxic properties, likely through apoptosis induction. In this study, we investigated its biological effects on HepG2 hepatocellular carcinoma cells, comparing its activity with the chemotherapeutic agent paclitaxel. We evaluated reactive oxygen species (ROS) production, membrane integrity, DNA damage, and wound closure using the scratch assay. At equivalent concentrations, PEGylated chrysin induced higher cell membrane damage but generated lower ROS levels compared to paclitaxel. Both compounds exhibited similar efficacy to reduce wound closure under the evaluated experimental conditions. Additionally, PEGylated chrysin significantly increased the frequency of micronuclei in binucleated cells, indicating genotoxic potential. To assess cellular uptake, a novel fluorescein−tagged conjugate (fluorescein−PEG−chrysin) was synthesized which demonstrated that the PEGylated drug is accumulated in the cytoplasm of HepG2 cells. These results highlight PEGylated chrysin as a promising candidate with antiproliferative potential, capable of inducing diverse cellular damage pathways in liver cancer cells while showing a distinct cytotoxic profile compared with Paclitaxel.

## Graphical abstract

PEGylated chrysin exhibited antiproliferative effects in HepG2 cells, inducing apoptosis, membrane damage, genotoxicity, and wound closure. Compared to paclitaxel, it caused lower ROS production and overall cytotoxicity, while efficiently accumulating in the cytoplasm, supporting its potential as a liver cancer therapeutic candidate.

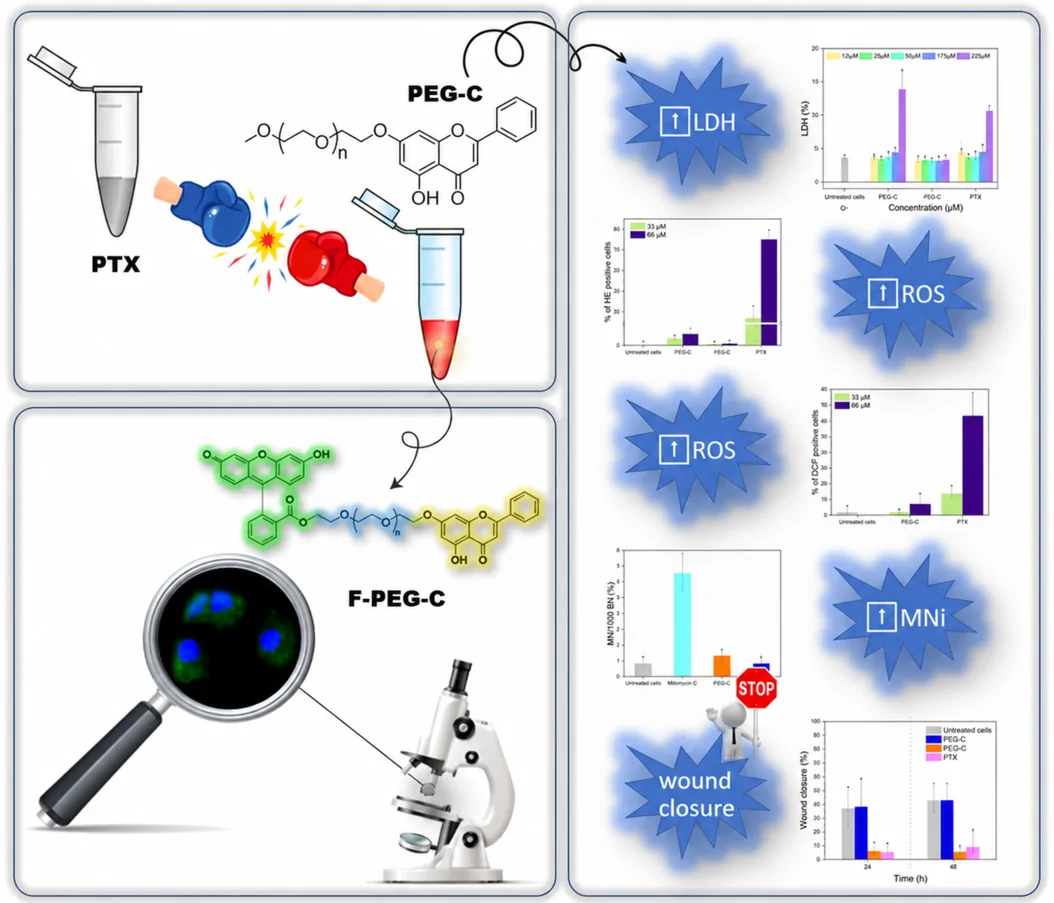

## 1 Introduction

Cancer is a common term for a large group of diseases characterized by the uncontrolled growth and proliferation of abnormal cells that invade and metastasize other parts of the body. It is one of the main causes of mortality and morbidity [1]. Among all types of cancer, liver cancer is a serious health problem and one of the leading causes of cancer−related mortality worldwide. Therefore, the design and evaluation of new anti−cancer drugs are of foremost pharmaceutical interest [1, 2].

Historically, natural products have been used for the investigation of innovative active pharmaceutical drugs. In connection, the identification of nutraceuticals and the modification of their structures to enhance their therapeutic effect, appear nowadays as a novel approach for the development of new anti−cancer therapies [3]. Although some natural products and nutraceuticals have low solubility and bioavailability, which limit their therapeutic value, these disadvantages can be surpassed using nanoscale delivery systems that increase the biological activity, both in vitro and in vivo [4]. Flavonoids are a large group of polyphenolic compounds synthesized as secondary metabolites by plants that have several favorable health effects [4, 5, 6]. Among flavonoids, chrysin is a natural flavone obtained from different sources, such as plants, honey and bee propolis, that would gain more medical and therapeutic relevance if some issues related to its low solubility and bioavailability are solved [4, 7]. Despite its low solubility and bioavailability, various recent studies have shown that chrysin has pro−apoptotic and antiproliferative properties against different types of cancer, indicating that this compound is a promising antitumoral agent amenable for further pharmaceutical development [8]. In line with this interesting progress, the mechanisms of action and the effect of chrysin on the metastatic potential of tumor cells are yet to be more thoroughly studied and understood [4, 9, 10].

The complexity of cancer is attributed to its multifactorial presentation, and the disruption of redox balance has been proposed to be one of the most important reasons underlying cancer development, progression and metastasis in human cells [11]. Thus, it has been reported that the disruption of redox homeostasis is a free radical induced process, in which ROS can play a central role for the control of migration and invasion processes of cancerous cells [12, 13]. In connection, it has been indicated that chrysin behaves as potent promoter of reactive oxygen species (ROS) production, which would play an important role in the induction of apoptosis as cell death mechanism [14, 15].

Paclitaxel (PTX), a natural chemotherapeutic agent isolated from Taxus brevifolia, is widely used against several types of solid tumors, such as breast, lung, colon, head and liver cancers [16]. However, the clinical efficacy of PTX is compromised by its poor water solubility, permeability and serious adverse effects [17]. In this sense, the search for new compounds that have lower capacity to generate side effects is relevant.

In a recent study we carried out the chemical conjugation of chrysin with low molecular weight methoxypolyethylene glycol (mPEG, 500 g/mol) in the C7 − OH of chrysin (PEGylated chrysin, PEG − C, Figure 1). Initial in vitro studies showed that this process does not compromise cytotoxic activity on PC3 and HepG2 tumor cells lines [18]. Aimed towards gaining a deeper understanding in the mechanism of action, in the present study ROS production, cell damage, and wound closure in a HepG2 tumor cell line treated with PEGylated chrysin were all evaluated, and the results were compared with those obtained with PTX. Additionally, the cellular uptake of PEG − C was qualitatively assessed using a fluorescein−labeled PEGylated derivative (Figure 1, F − PEG − C). Noteworthy, herein we demonstrate that PEGylation does not affect the in vitro activity of chrysin, further supporting the idea that PEG − C is a promising candidate for cancer treatment.

**Figure 1:**
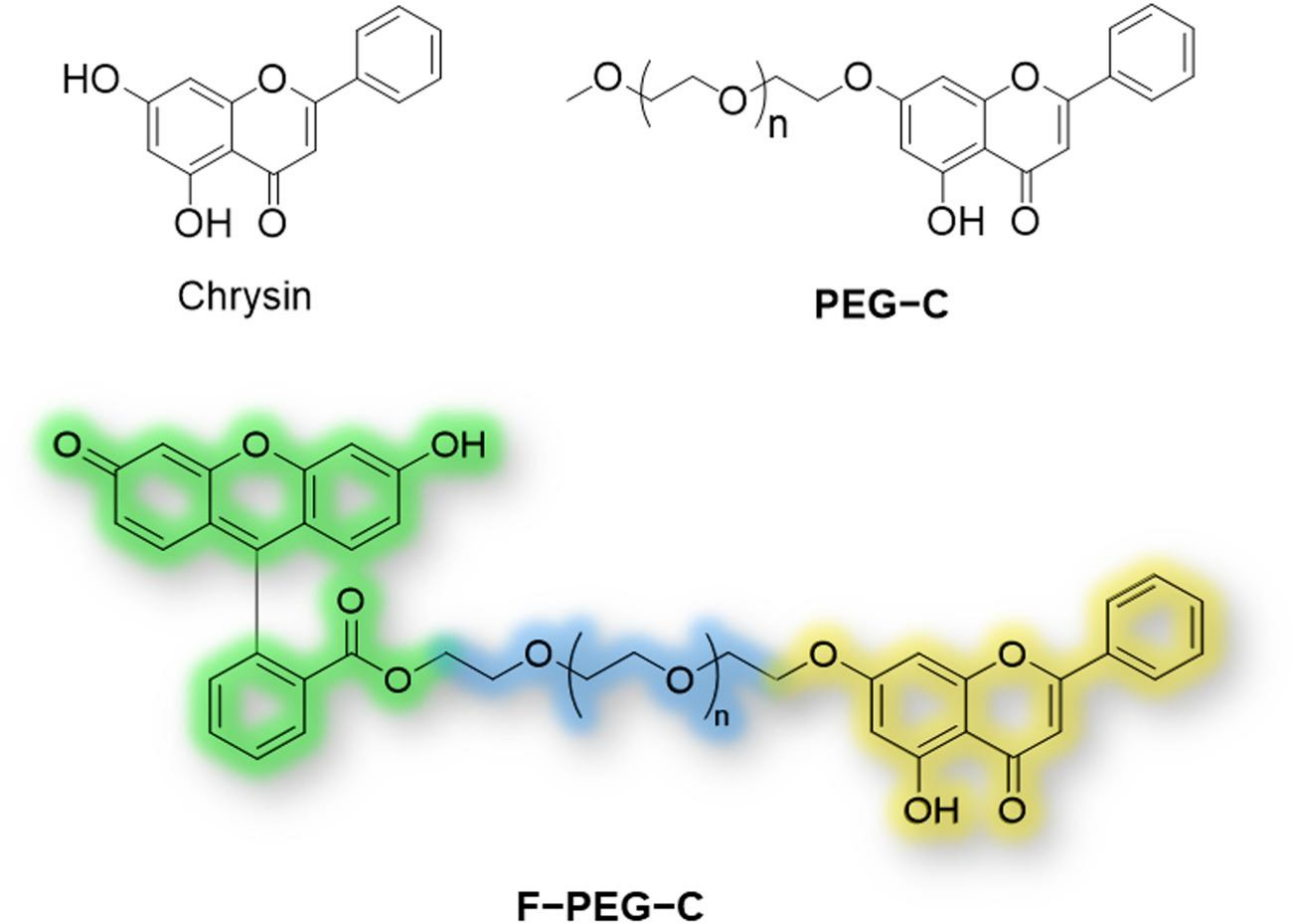
Structure of chrysin, PEGylated chrysin (PEG − C) and fluorescein−labeled PEGylated derivative (F − PEG − C).

## 2 Materials and Methods

### 2.1 Cell Culture and Reagents

HepG2 human hepatocellular carcinoma cells (ATCC HB − 8065. Hepatocellular Carcinoma, Human, Homo sapiens. Lot Number: 62591368 (12/31/2014). RRID: CVCL_0027) were cultured in DMEM − F12 medium (Gibco, USA) supplemented with 10% fetal bovine serum (FBS; Internegocios, Argentina) in a 5% CO_2_ humidified atmosphere at 37°C. This cell line exhibiting epithelial‐like morphology that was isolated from a hepatocellular carcinoma of a 15‐year‐old, White, male youth with liver cancer. The cell line used in this study was authenticated for the described experiments; it was purchased from ATCC and analyzed by PCR for *mycoplasma* contamination, confirming it was free of such contamination prior to conducting the experiments. 3 − [4,5−dimethylthiazol−2−thiazolyl]−2,5−diphenyltetrazolium bromide (MTT), dihydroethidium (HE) and 2′,7′−diclorodihidrofluorescein diacetate (DCFH − DA) were purchased from Invitrogen (Invitrogen, USA). The lactate dehydrogenase determination kit was purchased from Wiener Lab. (Rosario, Argentina). Cytochalasin−B was obtained from Sigma−Aldrich (USA). Giemsa solution was from Biopack (Argentina). Dimethyl sulfoxide (DMSO) was from Mp−Biomedicals (Argentina) and Triton X − 100 was from Biobasic (Argentina). Hydrogen peroxide and methanol were purchased to Cicarelli (Argentina). Mitomycin C was purchased to Microsules (Argentina). Paclitaxel (PTX, Fresenius Kabi) was kindly supplied by Lipomize S.R.L. (Parque Tecnológico del Litoral Centro, Santa Fe, Argentina). PEG − C was obtained as described by Oggero et al. [18] *N,N'*−dicyclohexylcarbodiimide, 4−dimethylaminopyridine and fluorescein were purchased to Sigma−Aldrich (USA).

### 2.2 Synthesis of Pegylated Chrysin (PEG − C) and PEGylated Chrysin − Fluorescein Conjugate (F − PEG − C)

The synthesis and characterization of PEG − C were carried out according to the methodology previously reported by our group. For the preparation of F − PEG − C, an analogous intermediate to PEG − C (HO − PEG − C) was first obtained from monotosylated PEG (400 g/mol) following the procedure described in Supporting Information (Figure S1). Copies of the ^1^H NMR spectra of PEG monotosylate and PEG‐C are provided in the Supporting Information (Figures S2 and S3, respectively). The esterification reaction was conducted under a nitrogen atmosphere. Fluorescein (1 mmol, 332.3 mg), *N,N'*−dicyclohexylcarbodiimide (DCC, 247 mg, 1.2 mmol), and 4−dimethylaminopyridine (DMAP, 33.6 mg, 0.275 mmol) were introduced into a 50 mL round−bottom flask equipped with a magnetic stirring bar, under inert atmosphere. HO − PEG − C (1.0 mmol, 654 mg), previously dissolved in DMF (10 mL), was then added to the mixture. The reaction was stirred in the dark for 24 h. After this period, additional portions of DCC (1.2 mmol, 247.5 mg) and DMAP (0.275 mmol, 33.6 mg) was added, and the reaction was allowed to proceed for 72 h. Reaction progress was monitored by thin−layer chromatography (dichloromethane: methanol 4:1; Rf ≈ 0.2). Upon completion, the crude mixture was filtered to remove the precipitated *N,N'−*dicyclohexylurea (DCU). Dichloromethane (50 mL) was added to the filtrate, and the organic phase was washed with water to remove unreacted fluorescein. The solvent was evaporated under reduced pressure, and residual DMF was removed by Kügelrohr distillation (70°C, 70 mmHg).

### 2.3 Spectroscopic Characterization of F − Peg − C

Spectroscopic characterization was performed using Fourier Transform Infrared Spectroscopy (Shimadzu FTIR − 8201 PC). PEG − C and F − PEG − C were dissolved in dichloromethane and deposited in a KBr window. The solvent was left to evaporate. Fluorescein was pulverized together with KBr and compressed into a slightly pink colored pellet. The spectra were acquired in the transmission mode (4000 cm^−1^ − 400 cm^−1^ range, scan number: 40). UV−Vis analyses were performed on a Shimadzu UV−Vis spectrophotometer (UV − 2401PC) in the range from 200 to 600 nm, in ethanol as solvent.

^1^H NMR experiments were obtained in DMSO−*d6* and CDCl_3_, in a Bruker Avance II 300 MHz spectrometer.

### 2.4 Cytotoxic Activity

#### 2.4.1 MTT Assay

Cells were seeded in 96−well plates at a density of 8000 cells per well. Cells were exposed for 48 h to different concentrations (13 µM to 265 µM) of mPEG (500 g/mol) and PTX. The concentration range used in this and subsequent assays was selected based on previous results obtained by our group [18]. In this study PTX was used as a positive control of toxicity and all assays were carried out in triplicate. Positive viability controls were also included, for which cells were incubated in culture medium and phosphate buffer (PBS, pH = 7), along with a negative viability control experiment, where cells were incubated with 5% hydrogen peroxide (Cicarelli, Argentina). To determine cell viability, 10 µL of MTT solution (5 mg/ml) was added to each well and the plates were incubated for 4 h at 37°C until the purple formazan crystals were formed. Subsequently, 100 µL of DMSO was added to each well to dissolve the formazan crystals.

Absorbance values (ABS) were recorded with a microplate reader (SPECTROstar Nano, BMG LABTECH, Germany) at a wavelength of 570 nm. Absorbance values were corrected with background absorbance, which was measured at 630 nm.

Cell viability (%) was calculated using Equation 1:

(1) %Viability=(ABSsample/ABSmedia)*100

Where ABS of the sample is the absorbance of the treated cells and the ABS media is the absorbance of the cells in the culture medium.

The evaluation of the viability of PEG − C through the MTT assay in HepG2 cells within the same range of concentrations was previously published [18].

#### 2.4.2 LDH Leakage Assay

The integrity of the cell membrane was assessed following exposure to PEG − C, mPEG, and PTX within a concentration range of 13 to 265 µM, measuring the leakage of lactate dehydrogenase (LDH) into the culture medium. Culture medium (100 µL) was aspirated and LDH activity in the medium was determined using a commercially available kit from Wiener Lab (LDH − P UV liquid line, Argentina), following the instructions of the manufacturer. The assay is based on the conversion of lactate to pyruvate in the presence of LDH with parallel reduction of NAD. The formation of NADH from the above reaction results in a change in the absorbance at 340 nm. The absorbance was measured using an automatic analyzer system (Wiener Lab CM250, Argentina). Cells incubated with culture medium and Triton X − 100 were used as negative and positive controls, respectively.

The percentage of LDH released from treated cells into the culture was calculated by comparison with the maximal amount released by cells lysed with 1% Triton X − 100 (positive control), according to the method described by Kumar et al. [19], and using Equation 2:

(2) %LDH(Ul/L)=(LDHsample/LDHTritónX−100)∗100

#### 2.4.3 ROS Production

Hydroethidine (HE) is a probe used for the detection of intracellular ROS (mainly by superoxide anion) [20]. HE, a reduced form of ethidium (Etd + ), is rapidly taken up by living cells and oxidized to Etd+ by ROS in a dose−dependent manner, which then intercalates into nucleic acids and becomes fluorescent [21]. In parallel, intracellular ROS production was also evaluated using 2',7'−dichlorodihydrofluorescein diacetate (DCFH − DA), a cell−permeable probe that is deacetylated intracellularly and oxidized by ROS to the fluorescent compound dichlorofluorescein (DCF) [22]. Briefly, 8000 cells per well were cultured in 96−well culture plates for 24 h. The cells were exposed to PEG − C, mPEG and PTX at concentrations of 33 and 66 µM for 48 h. These concentrations, used in this and subsequent assays, correspond to the IC50/4 and IC50/2 values, respectively, and were previously determined by our group. Cells incubated with hydrogen peroxide were used as positive control. After treatment, cells were centrifuged at 800 x g for 5 min. The supernatant was discarded and the cells were incubated with 100 µL of a 0.1 µM DHE solution for 15 min at 37°C in darkness. For DCFH − DA analysis, cells were incubated with 10 µM DCFH − DA solution under the same conditions. Finally, the cells were centrifuged at 800 × g for 5 min, resuspended in focusing fluid and analyzed by flow cytometry (Attune, NxT Acoustic Focusing Cytometer A24860, Life Technology, Carlsbad, CA, USA). Data were collected and analyzed using the FlowJo software (FlowJo software. BD Biosciences. USA. RRID: nif−0000 − 00280, https://www.flowjo.com/).

### 2.5 Wound Healing Assay

The wound healing assay was used to evaluate wound closure [23]. Cells were seeded in 24−well plates and allowed to reach 90% confluency. Monolayers were scraped in the center of the wells with pipette tips and washed with PBS to remove cellular debris. Then, cells were cultured in DMEM − F12 medium without FBS in the presence or absence of PEG − C, mPEG and PTX at a concentration of 33 µM for 48 h. Wound infliction was considered as time 0, and wound closure was monitored for up to 48 h. Cell migration towards the wound area was documented at 0, 24, and 48 h using a Zeiss Axio Observer microscope (Zeiss). The area covered by migrating cells was quantified using ImageJ software, and the percentage of wound closure was calculated using Equation 3, where *t* = Xh corresponds to the evaluated time.

(3) Woundclosure%=(At=0–At=Xh/At=0)*100

### 2.6 In Vitro cellular uptake

To study the cellular uptake of PEG − C in HepG2 cells, PEG − C was labeled with fluorescein as a fluorescent marker as was described earlier (F − PEG − C). HepG2 cells were seeded at a density of 150,000 cells per well in 12−well plates containing coverslips and incubated for 24 h. The cells were then treated with F − PEG − C (33 µM) for 0, 24 and 48 h. HepG2 cells treated with free FITC served as control group. The nuclei of the cells were labeled with Hoechst 33342 (1:1000 dilution) (ThermoFisher) for 10 min. Subsequently, the cells were washed with PBS 1X and fixed with 1% paraformaldehyde for 15 min. The intracellular uptake of the conjugate was analyzed using an epifluorescence microscope at 20X magnification (Zeiss Axio Observer) and confocal microscope at 40X magnification (Carl Zeiss confocal LSM 880). To prevent photobleaching all experimental steps were carried out under light−protected conditions.

### 2.7 Cytokinesis−Block Micronucleus (CBMN Assay)

DNA damage was determined by in vitro micronucleus assay (CBMN). The CBMN assay was performed following the protocol of Fenech et al. [24], with some modifications and according to guideline 487 of the Organization for Economic Cooperation and Development (OECD) [25]. Briefly, HepG2 cells were seeded in 96−well plates at a density of 8000 cells per well. After exposure for 48 h to PEG − C and mPEG at a concentration of 33 µM, the medium was replaced with DMEM − F12 supplemented with 10% FBS containing cytochalasin B (3 µg/mL) and cells were incubated for 20 h. Cells incubated with Mitomicyn C (0.25 µg/mL) were used as a positive control. After cytochalasin B treatment, cells were washed with PBS. Aliquots of 100 µL of cell suspension were transferred to cytocentrifuge cups and centrifuged for 5 min at 700 rpm to produce one spot per slide. The slides were then removed, fixed with methanol and stained with Giemsa. The samples were observed under the Zeiss Axio Observer microscope, and 10 photographs were taken at random. Micronuclei (MNi) were scored in binucleated (BN) cells and a total of 1000 BN cells per each experimental point were scored to determine the parameters of CBMN assay. All data were obtained from three independent experiments. The percentage of MNi per 1000 BN cells (%MNBN) was determined using the Equation 4.

(4) %MNBN=(MNBN/1000BN)*100

### 2.8 Statistical Analysis

Data were analyzed using SPSS 22.0 statistical software for Windows (SPSS Inc., Chicago, IL, USA). Data normality was performed using the Shapiro−Wilk test. For the LDH assay, a two−way analysis of variance (ANOVA) was performed to evaluate the effects of concentration and treatment, as well as their interaction. This analysis was followed by Tukey's HSD post hoc test for multiple comparisons between groups. A *p* value < 0.05 was considered statistically significant. The results were expressed as mean ± standard deviation (SD). For HE and DFC production, micronucleus frequency, and percent wound closure assays, differences between groups were analyzed using one−way ANOVA followed by Tukey's HSD post hoc test. A *p*−value < 0.05 was considered statistically significant. Results are expressed as mean ± standard deviation (SD).

## 3 Results

### 3.1 Chemistry

The fluorescein−labeled PEG − C conjugate was synthesized through a multistep strategy outlined in Figure 2. In the first step, the heterobifunctional intermediate HO − PEG − C was obtained from monotosylated PEG (400 g/mol) and chrysin. The intermediate was isolated in high yield, purified by column chromatography, and characterized by ^1^H NMR spectroscopy (see Figure S3). In the second step, HO − PEG − C was reacted with fluorescein under Steglich esterification conditions. Conversion of the starting OH–PEG–C was nearly quantitative, and the labeled conjugate was obtained with adequate purity for its use in cellular uptake studies.

**Figure 2:**
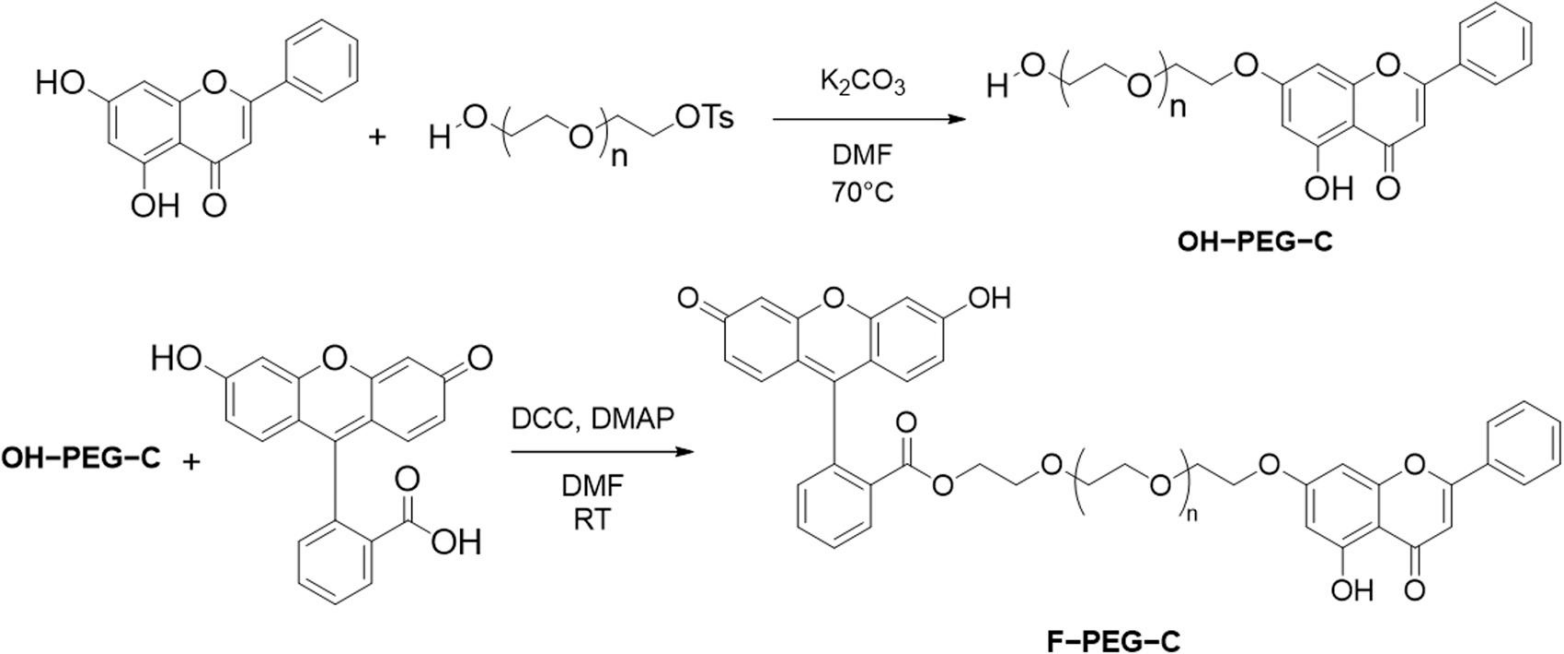
Synthetic route to F − PEG − C.

F − PEG − C was characterized by ^1^H NMR spectroscopy (Figure 3). After the esterification reaction, the signal corresponding to the terminal hydroxyl proton of the PEG chain at 4.60 ppm (1H, singlet) disappeared, and a signal appeared at 4.58 ppm (2H, triplet), which is consistent with the formation of the ester bond. The characteristic signals of the chrysin and fluorescein cores were retained, confirming that no structural modifications occurred outside the esterification site. Figure 4a shows the FTIR spectra of fluorescein, PEG − C, and the F − PEG − C conjugate. PEG–C exhibits characteristic absorption bands at 1655 cm^−1^ (*ν* C = O), 1623 and 1485 cm^−1^ (*ν* C = C), and 1357 cm^−1^ (*ν* C = C + *ν* C–O). Fluorescein displays absorption bands at 3315 cm^−1^ (*ν* OH), 3050 cm^−1^ (*ν* X = CH), 1580 cm^−1^ (*ν* C = O), and additional aromatic signals at 1458, 1393, 663, and 597 cm^−1^. Upon conjugation, a new band appears at 1720 cm^−1^, assigned to the ester *ν* C = O stretching vibration, while the band at 1690 cm^−1^ —characteristic of the free carboxyl group of fluorescein—disappears. Notably, the signals associated with PEG‐C remain unchanged, indicating that conjugation selectively occurs at the carboxylic group of fluorescein. Figure 4b presents the UV–Vis absorption spectra of PEG–C, fluorescein, and the F − PEG − C conjugate in ethanol. PEG–C shows two characteristic absorption maxima at 272 and 316 nm, corresponding to the flavonoid chromophore of chrysin. Fluorescein displays a strong absorption band around 492 nm and an additional band in the ultraviolet region (256–265 nm) associated with π → π* transitions. In the spectrum of F − PEG − C, both PEG − C and fluorescein absorption features are preserved, confirming incorporation of both components into the final conjugate. The absorption peak around 450 nm closely matches that of the free fluorescein chromophore, indicating that the labeling process does not significantly alter its electronic properties.

**Figure 3:**
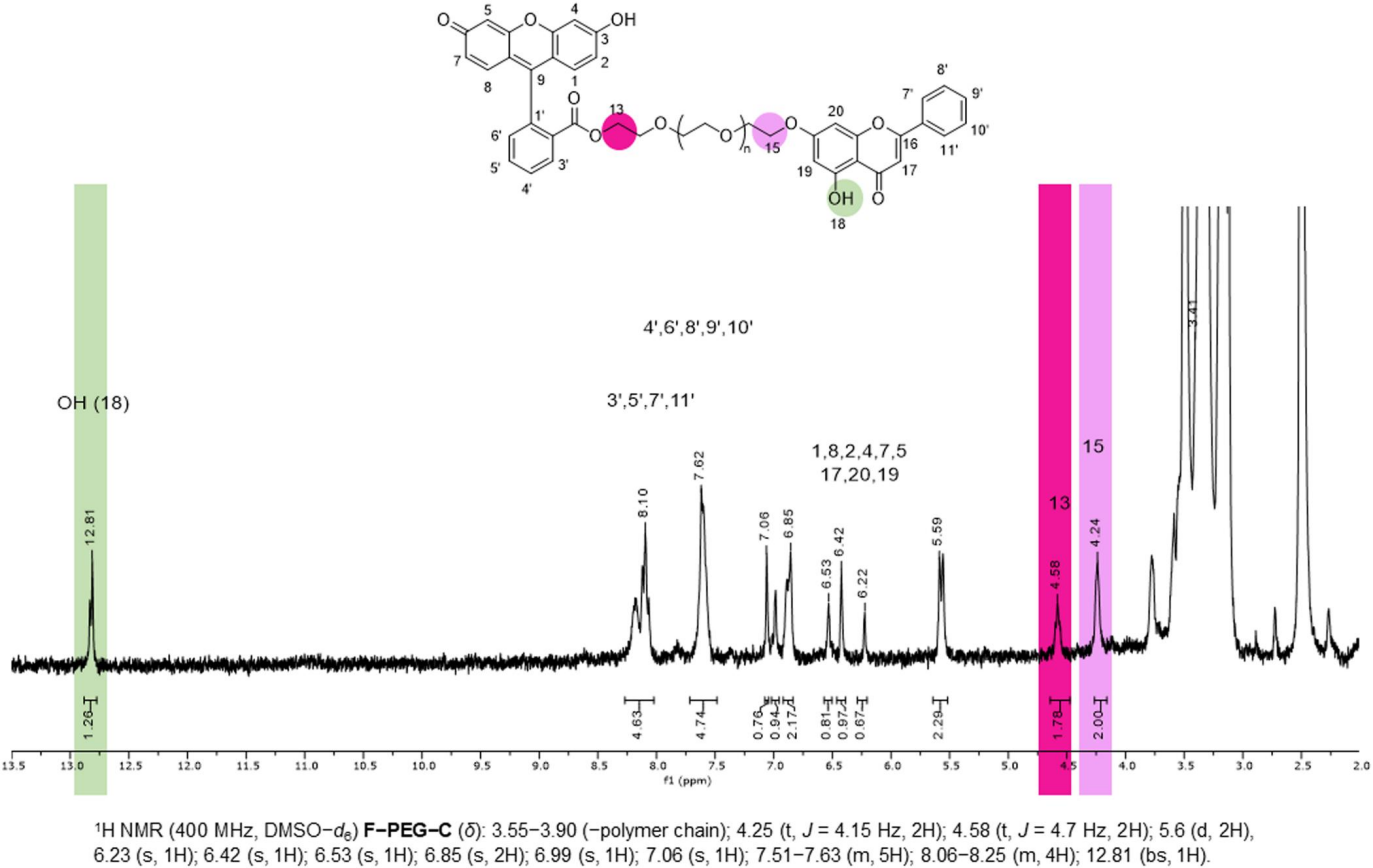
^1^H NMR spectra for F − PEG − C.

**Figure 4:**
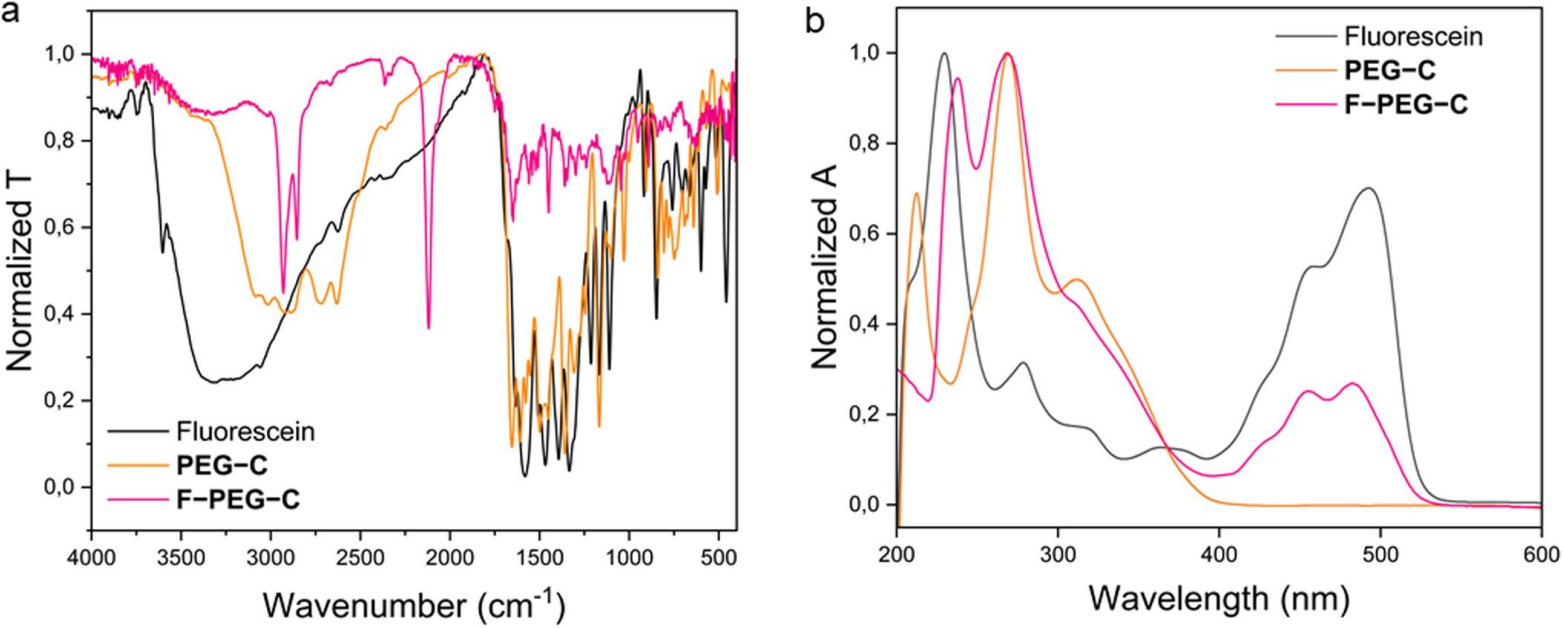
a) FTIR and b) UV−vis spectra for fluorescein, PEG − C and F − PEG − C.

### 3.2 Aqueous Solubility of PEG − C

The aqueous solubility of native chrysin and the PEG − C conjugate was determined in neutral aqueous media [26] and in phosphate buffer saline (PBS 0.1 M, pH: 7.4). Native chrysin exhibited a very low solubility of 2 × 10^−3^ mM, corresponding to 5.08 × 10^−4^ mg of chrysin/mL at pH: 7. In contrast, PEG − C showed a markedly higher solubility of 20 mM, equivalent to 15.08 mg chrysin/mL. Additionally, solubility measurements performed in PBS yielded comparable values for PEG − C, showing solubility values of 20 mg/mL in equivalent to chrysin. In contrast, chrysin is known to be poorly soluble under similar conditions, with reported values of 0.06 mg/mL in PBS [27]. Based on this comparison, PEGylation results in an increase in solubility of approximately four orders of magnitude.

### 3.3 Cytotoxic Activity

The antiproliferative or cytotoxic effects of PEG − C, mPEG, and PTX were analyzed using the MTT assay. As previously described by our group, PEG − C and chrysin inhibit cell proliferation of the HepG2 cell line in a concentration−dependent manner. As expected, in the present study we found that PTX inhibited cell proliferation in a concentration−dependent manner too, while mPEG did not inhibit the proliferation of the HepG2 cell line at any of the evaluated concentrations (see Figure S4). According to ISO standards [28], biopharmaceuticals are considered to be cytotoxic when they can reduce cell viability to 70% or less. In our previous study, PEG − C inhibited the proliferation of HepG2 cell line at concentrations ranging from 66 to 265 µM, while in this study, PTX inhibited HepG2 cell line proliferation at concentrations ranging from 33 µM to 265 µM, after 48 h.

Cell membrane damage induced by PEG − C, mPEG and PTX was evaluated by LDH release assay. Cells treatment with the tested concentrations range (13–265 µM) of PEG − C and PTX resulted in significantly higher LDH release at a concentration of 265 µM compared with the untreated cells (*p* < 0.05). In contrast, cell treatment with mPEG at all concentrations showed no difference in LDH release compared to untreated cells (*p* > 0.05) (Figure 5).

**Figure 5:**
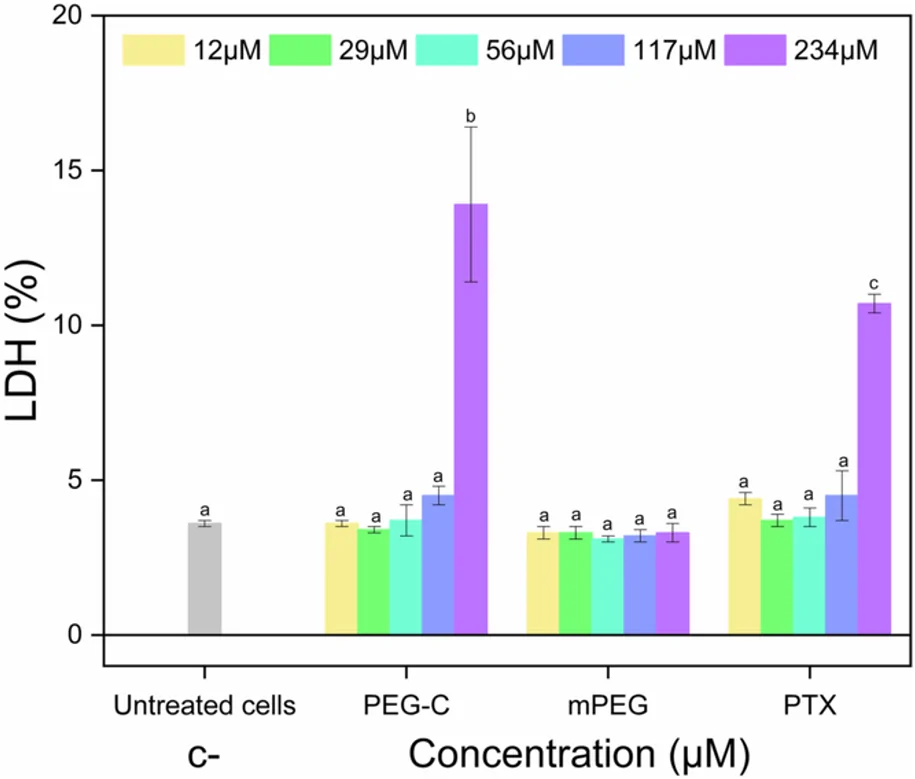
Lactate dehydrogenase (LDH) activity was measured in the culture medium collected after HepG2 cell treatment with different concentrations of PEG − C, mPEG, and PTX. Error bars represent standard deviation (SD). Statistical analysis was performed using two−way ANOVA, revealing significant effects of treatment (*p* < 0.001), concentration (*p* < 0.001), and their interaction (*p* < 0.001). Tukey's HSD post hoc test was used for multiple comparisons. Different letters indicate statistically significant differences between groups (*p* < 0.05). C− represents the negative control (untreated cells).

The percentages of HepG2 that produced superoxide (HE positive cells) were higher for cells treated with PEG − C and PTX (in a dose−dependent manner) than the percentages for untreated cells (*p* < 0.05). Conversely, the percentage of HepG2 cells that produced superoxide in cells treated with mPEG at all concentrations was similar to that of untreated cells (*p* > 0.05) (Figure 6A). Also, the percentages of HepG2 cells that produced ROS (DCF positive cells) were higher for cells treated with PEG − C and PTX than the percentages for untreated cells (*p* < 0.05) (Figure 6B). In addition, PTX treatment induced the highest levels of ROS production among all experimental conditions evaluated.

**Figure 6:**
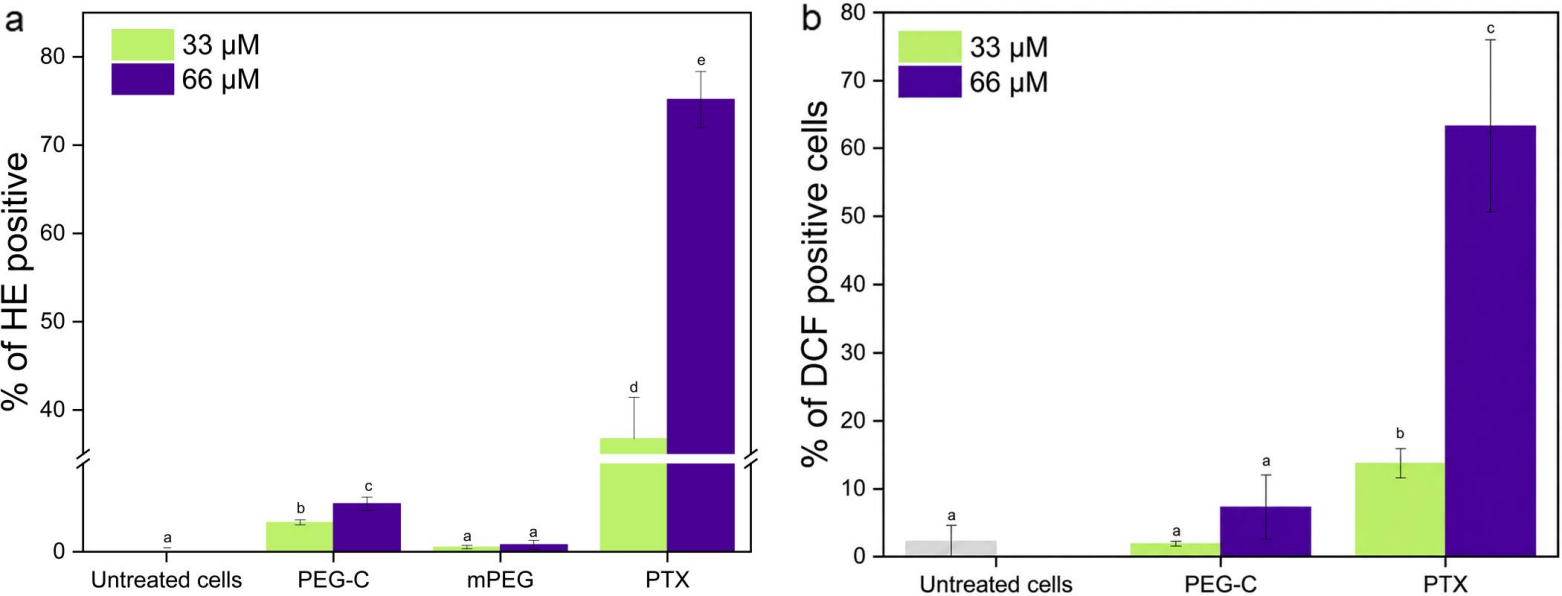
(A) Percentage of HepG2 cells that produce superoxide (% HE positive cells) exposed to PEG − C, mPEG and PTX at 33 and 66 µM, respectively. (B) Percentage of HepG2 cells that produce Intracellular ROS (% DCF positive cells) exposed to PEG − C and PTX at 33 and 66 µM. Bars represent the mean ± SD of 3 independent experiments. Different letters indicate significant difference.

### 3.4 Cell Wound Closure Assay

Wound closure was evaluated using the scratch wound healing assay. The results showed that the percentage of wound closure was lower after treatment with PEG − C and PTX, at both 24 and 48 h, than in untreated cells and mPEG−treated cells (*p* < 0.05). (Figure 7).

**Figure 7:**
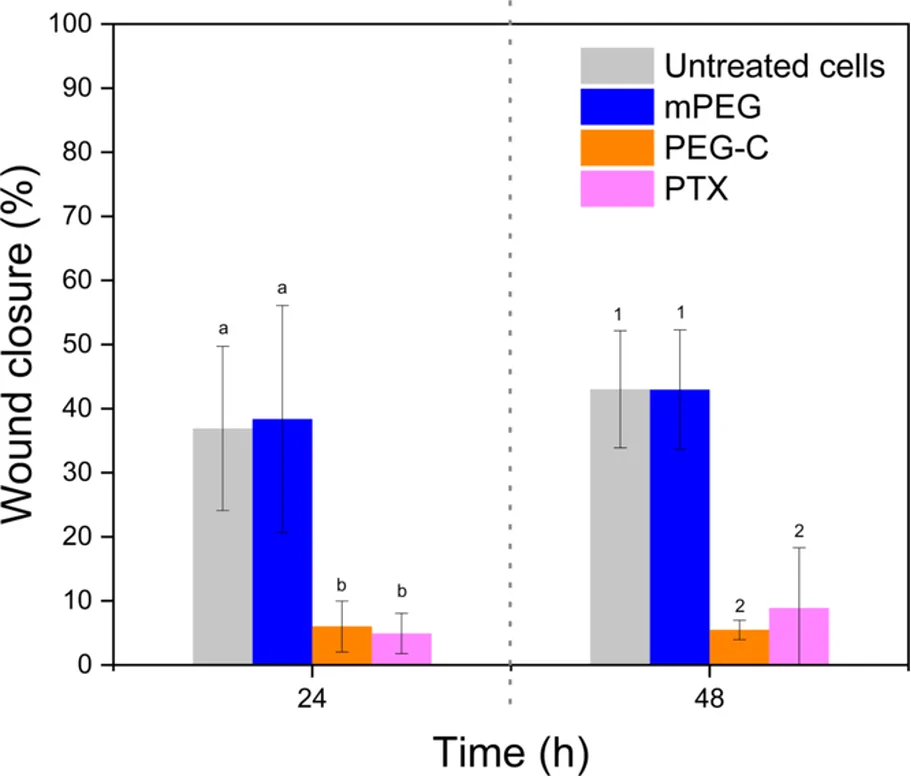
Percentage of wound closure in HepG2 cells. Bars represent the mean ± SD of 3 independent experiments. Different letters and numbers indicate significant difference (*p* < 0.05).

### 3.5 In Vitro cellular uptake F − PEG − C

The cellular uptake of F − PEG − C was qualitatively evaluated in the HepG2 cell line. The results demonstrated that treated cells exhibited green fluorescence in the cytoplasm, confirming the internalization of the compound (Figure 8).

**Figure 8:**
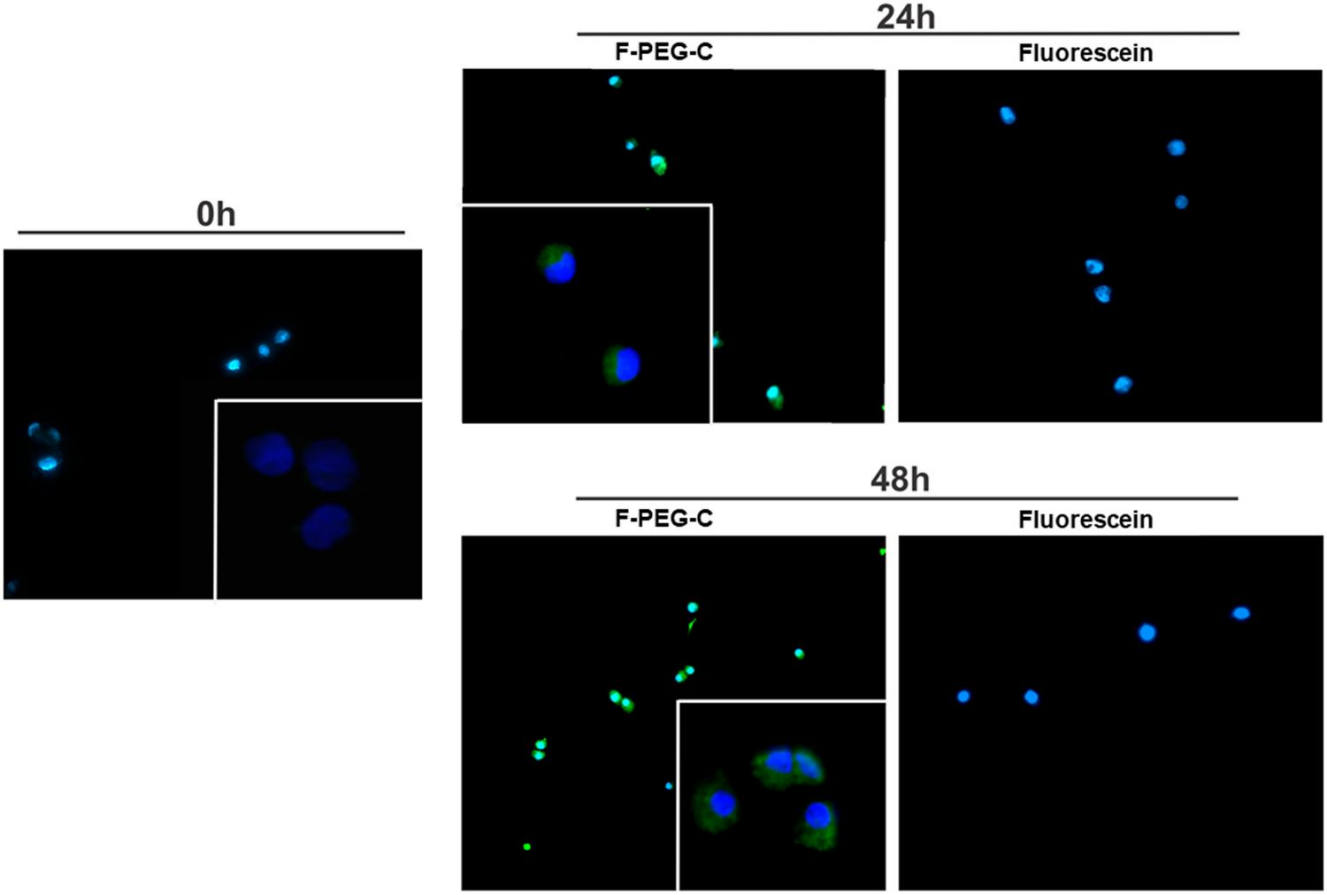
Cellular uptake of Fluorescein and F − PEG − C in HepG2 cells at different time points. Nuclei were counterstained with Hoechst 33342 (blue fluorescence). Fluorescein: green fluorescence.

### 3.6 Cytokinesis−Block Micronucleus (CBMN Assay)

The ability of PEG − C and mPEG to induce chromosomal and genomic damage in HepG2 cells was investigated using a cytokinesis−blocking micronucleus assay. After 48 h of treatment, an increase in the % of MNi in binucleated cells treated with PEG − C compared to untreated cells was observed (*p* < 0.05). No difference was observed in cell treated with mPEG compared to untreated cells (*p* > 0.05). Mitomycin C, used as positive control, afforded a significantly higher % of MNi than PEG − C, mPEG treated cells and untreated cells (*p* < 0.05) (Figure 9).

**Figure 9:**
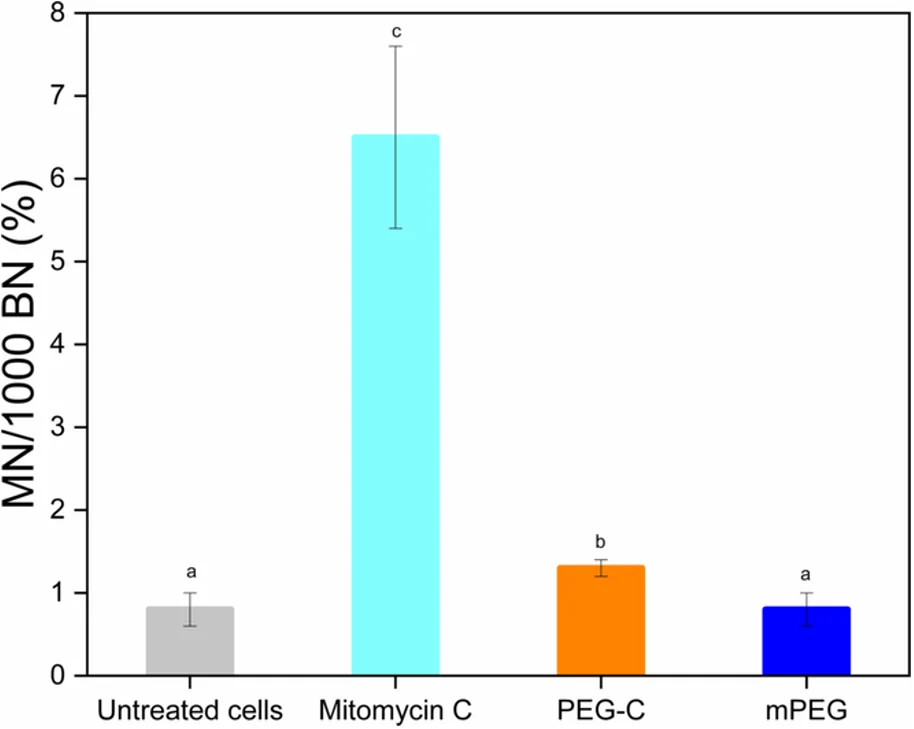
Micronucleus frequency (% of MNi/1000 binucleated cells) in HepG2 cells exposed to PEG − C and mPEG for 48 h assessed by CBMN assay. Mitomycin C was used as a positive control. Data represent mean ± SD. Different letters indicate significant difference.

## 4 Discussion

Chrysin is a naturally occurring flavonoid with well−documented antitumor activity across a wide range of cancer models [29, 30, 31, 32, 33, 34]. Consistent with our previous findings, PEGylation of chrysin did not significantly alter its intrinsic cytotoxicity toward HepG2 cells, supporting that PEGylation preserves the bioactive pharmacophore. Paclitaxel (PTX), a well−established chemotherapeutic drug, was used as a positive control due to its robust cytotoxicity. The primary mechanism of action of PTX involves the excessive stabilization of microtubule dynamics, which disrupts normal mitotic progression and ultimately impairs cell division. In addition to its antimitotic activity, PTX can also interfere with various microtubule−dependent cellular processes, such as intracellular transport, signaling pathways, and organelle movement. Furthermore, recent studies have demonstrated that PTX promotes the early generation of reactive oxygen species (ROS) in tumor cells, and hydrogen peroxide plays a significant role in PTX−induced cell death both in vitro and in vivo [35, 36]. However, despite its efficacy, PTX is associated with dose−limiting toxicity, hypersensitivity, and metabolic instability [37], underscoring the need for alternative molecules with safer toxicity profiles. In this context, exploring PEGylated chrysin (PEG–C) is particularly relevant. Assessment of cytotoxicity through complementary assays revealed differential sensitivity between methods. LDH release, which reflects loss of membrane integrity, was only evident at the highest concentration of PEG–C and PTX, whereas the MTT assay, which reflects mitochondrial metabolic activity, detected significant reductions in viability at lower concentrations. These observations align with the mechanistic difference between the assays: membrane disruption represents a late−stage event, while mitochondrial dysfunction often constitutes an early hallmark of cell injury [38]. Thus, MTT provides a more sensitive indicator of early cytotoxicity in HepG2 cells, supporting the involvement of mitochondrial impairment as an initiating event in PEG–C–mediated cell death. In line with these findings, both PEG–C and PTX induced a concentration−dependent increase in intracellular ROS. ROS are chemically reactive molecules generated as natural by−products of cellular metabolism, primarily in the mitochondria during oxidative phosphorylation. Under physiological conditions, low levels of ROS play important roles in cell signaling, immune responses, and the regulation of gene expression. However, excessive ROS production or insufficient antioxidant defense leads to a condition known as oxidative stress. This imbalance can damage essential biomolecules including lipids, proteins, and DNA, thereby disrupting cellular structure and function. Lipid peroxidation caused by ROS compromises membrane integrity, while oxidative modifications of proteins can alter enzyme activity and signaling pathways. Furthermore, oxidative DNA damage may contribute to mutations and genomic instability. To counteract these effects, cells rely on an antioxidant defense system composed of enzymatic antioxidants such as superoxide dismutase, catalase, and glutathione peroxidase, as well as non−enzymatic antioxidants including glutathione, vitamins C and E, and trace elements. When this protective system becomes overwhelmed, oxidative stress can trigger inflammation, apoptosis, and various pathological conditions, highlighting the importance of maintaining redox homeostasis for normal cellular function [39, 40, 41, 42]. Our observations are consistent with previous work showing that chrysin may promote apoptosis in diverse cancer models via ROS−mediated mitochondrial pathways [34, 43, 44, 45]. In a previous study conducted by our research group, apoptosis was evaluated by Annexin V/PI staining and flow cytometry, suggesting early apoptosis−related effects associated with PEGylated chrysin exposure in HepG2 cells. Importantly, PEG − C produced ROS levels sufficient to elicit cytotoxicity but notably lower than PTX, suggesting a potentially diminished risk of oxidative side effects. Given that cancer cells exhibit upregulated ROS metabolism and are particularly vulnerable to redox imbalance [46, 47, 48], PEG − C appears to exploit this selective vulnerability.

The increased frequency of micronuclei observed after exposure to PEG − C indicates the induction of chromosomal instability and/or mitotic alterations. While genotoxicity is a significant safety concern, it must be interpreted within the context of anticancer drug development, as many conventional chemotherapeutic agents exert their therapeutic effects through mechanisms related to DNA damage. Consistent with this, DNA integrity analysis demonstrated that PEG − C induces measurable genotoxic stress, supporting ROS−dependent DNA damage as a mechanism contributing to its antitumor activity. Similar effects have been described for chrysin, which promotes micronucleus formation, chromosomal alterations, and DNA strand breaks in various cancer cell lines [49, 50, 51]. Given that DNA damage and repair pathways are fundamental to the efficacy of many anticancer agents [52, 53], PEG − C−induced oxidative lesions could contribute to its cytotoxic effects. Notably, PEG − C produced a lower level of DNA damage compared to mitomycin C, suggesting a potentially more favorable risk−benefit balance compared to conventional treatments. In this sense, therapy−related cancers are secondary primary malignancies that develop as a consequence of exposure to genotoxic chemotherapeutic agents and/or radiotherapy, representing a distinct group of neoplasms with a well−established etiological origin [54].

However, further studies are needed to better characterize the type and extent of DNA damage before in vivo evaluation. In this regard, complementary approaches such as the comet assay and γH2AX detection would provide valuable information on DNA strand breaks and early responses to DNA damage, allowing for a more comprehensive assessment of genotoxic profile of PEG − C [17, 55, 56].

The scratch wound healing assay showed that PEG‐C significantly reduced wound closure compared with untreated cells. This reduction was comparable to that observed with PTX, suggesting that PEGylation does not diminish the ability of chrysin to interfere with the wound closure process under the evaluated experimental conditions. These findings are consistent with previous studies reporting anti‐migratory and anti‐invasive effects of chrysin and its derivatives in different cancer models under both normoxic and hypoxic conditions [32, 57, 58]. However, the scratch assay does not distinguish between reduced cell motility and delayed wound closure resulting from decreased cell viability and/or proliferation. Therefore, although the assay was performed under serum‐free conditions to minimize cell proliferation, the contribution of PEG‐C‐induced cytotoxicity to the reduced wound closure cannot be completely excluded. Consequently, the present findings should not be interpreted as direct evidence of a specific anti‐migratory effect. Future studies using complementary approaches, such as transwell migration assays or other proliferation‐independent migration assays, will be necessary to determine whether PEG‐C directly affects cell motility. Given the importance of cell migration in metastatic dissemination, these additional studies will help clarify the potential of PEG‐C to interfere with metastatic progression.

Fluorescence effects imaging using a F − PEG − C conjugate demonstrated clear intracellular accumulation with a time−dependent increase in cytoplasmic localization. Recent work [59] indicates that small PEG molecules (750–2000 g/mol) may cross cellular membranes predominantly via passive diffusion rather than endocytosis, thereby enabling efficient intracellular access. Our observations support this mechanism, providing direct evidence for the capacity of low−molecular−weight PEG conjugates to penetrate cells and accumulate over extended incubation times. This property is particularly advantageous for the delivery of hydrophobic flavonoids such as chrysin.

Although HepG2 cells successfully internalized PEG‐C after 48 h of incubation in complete culture medium, as demonstrated by fluorescence microscopy, and retained their biological activity in the wound closure assay performed under serum‐free conditions, the physicochemical stability of the conjugate in DMEM supplemented with 10% FBS was not specifically evaluated. Therefore, the potential influence of interactions with serum proteins on the behavior of PEG‐C, including its cellular uptake and biological activity, cannot be ruled out. Future studies should investigate the stability and physicochemical properties of PEG‐C in biologically relevant media to better understand its interactions with serum components and their potential impact on its biological performance.

Our results demonstrate that PEG‐C retains the key bioactivities of chrysin, namely mitochondrial impairment, ROS generation, and genotoxic stress, while potentially reducing the extent of oxidative damage compared with PTX. These attributes, together with its improved solubility and straightforward synthesis, position PEG‐C as a promising lead structure for the development of flavonoid‐based anticancer agents. The concentrations evaluated in this study were selected based on the previously determined IC_50_ of PEG‐C in HepG2 cells after 48 h of exposure allowing the mechanistic assays to be performed at biologically relevant concentrations associated with measurable cytotoxic effects. Although the pharmacokinetic profile of PEG‐C has not yet been established, native chrysin is known to exhibit extremely low oral bioavailability owing to its poor aqueous solubility, extensive phase II metabolism, and rapid elimination [27]. Therefore, PEGylation was designed not only to improve the aqueous solubility of chrysin but also to potentially overcome some of these pharmacokinetic limitations. Nevertheless, dedicated pharmacokinetic and biodistribution studies will be required to determine whether therapeutically relevant concentrations of PEG‐C can be achieved in vivo and to establish the translational relevance of the present findings.

In addition, its selectivity toward tumor cells and safety profile remain to be established, as the present study was conducted exclusively in a hepatocellular carcinoma cell line. Future studies should evaluate its pharmacokinetics, biodistribution, therapeutic efficacy in in vivo tumor models, and safety in normal cell models and animal studies to better define its therapeutic window and determine its suitability for further preclinical development.

## 5 Conclusion

In this study, we demonstrated that PEGylation of chrysin does not compromise its in vitro biological activity. PEGylated chrysin exhibited both antiproliferative and cytotoxic effects against the human hepatoma cell line HepG2. The observed activity appears to be mediated through multiple mechanisms, including cell membrane disruption, DNA damage, and oxidative stress, which would ultimately lead to apoptosis. Notably, PEG − C induced less DNA damage than PTX, suggesting a potentially safer genotoxic profile. In addition, PEG − C effectively reduced wound closure in the scratch assay and was successfully internalized by HepG2 cells, as confirmed by fluorescence microscopy using a fluorescein−labeled derivative. Taken together, these findings highlight PEG − C as a promising antitumor candidate that retains efficacy while potentially reducing toxicity. Further preclinical studies are warranted to fully assess its therapeutic potential and safety profile.

## Author Contributions

**Fátima Belén Gasser:** conceptualization, investigation, writing – original draft, methodology. **Julia Oggero:** conceptualization, investigation, writing – original draft, methodology. **María Sol Renna:** methodology, writing – original draft. **María Florencia Beltramino:** methodology, writing – original draft. **Candela Simonetto:** methodology, writing – original draft. **Lucía Dominguez:** methodology. **Hugo Héctor Ortega:** writing – review and editing. **Santiago Eduardo Vaillard:** writing – review and editing. **Victoria Anahí Vaillard:** conceptualization, investigation, visualization, writing – review and editing, supervision, resources. **María Eugenia Baravalle:** conceptualization, investigation, visualization, writing – review and editing, supervision, resources.

## Conflicts of Interest

The authors declare no conflicts of interest.

## Declaration of Generative AI Use

During the preparation of this work, the authors used Notebook (Google) and ChatGPT to assist with English language refinement. After using this tool, the authors reviewed and edited the content as needed and take full responsibility for the content of the published article.

## Supporting information


Supporting File

## Data Availability

The data used to support the findings of this study are available from the corresponding author upon request.
