# Supplementary material for: PEGylated Chrysin: In Vitro Biological Activity and Mechanistic Insights in HepG2 Cells
Source: J Biochem Mol Toxicol. 2026 Nov 4;40(11):e71169. doi: 10.1002/jbt.71169 (PMC13734287; doi:10.1002/jbt.71169)
Supplement: Supplementary file 1 [file JBT-40-e71169-s001.docx]

**Supporting information**

**PEGylated Chrysin: In Vitro Biological Activity and Mechanistic Insights in HepG2 Cells**

**Fátima Belén Gasser^1^, Julia Oggero^2^, María Sol Renna^1^, María Florencia Beltramino^1^, Candela Simonetto^1^, Lucía Dominguez^3,^ Hugo Héctor Ortega^1^, Santiago Eduardo Vaillard^2^, Victoria Anahí Vaillard^2*^, María Eugenia Baravalle^1,4*^**

^1^Centro de Medicina Comparada, Instituto de Ciencias Veterinarias del Litoral (ICIVET). Universidad Nacional del Litoral and Consejo Nacional de Investigaciones Científicas y Técnicas (CONICET). Esperanza, Santa Fe, Argentina.

^2^Instituto de Desarrollo Tecnológico para la Industria Química (INTEC). Universidad Nacional del Litoral and Consejo Nacional de Investigaciones Científicas y Técnicas (CONICET). Santa Fe, Argentina

^3^Laboratorio de Microscopia Aplicada a Estudios Moleculares y Celulares (LAMAE). Instituto de Investigación y Desarrollo en Bioingeniería y Bioinformática (IBB), UNER. Oro Verde, Entre Ríos, Argentina.

^4^Centro Universitario Gálvez. Universidad Nacional del Litoral (UNL), Gálvez, Santa Fe, Argentina.

***** Baravalle, M.E. and Vaillard, V.A. should be considered joint senior authors.

Corresponding author: Baravalle, M.E. Centro de Medicina Comparada, Instituto de Ciencias Veterinarias del Litoral (ICIVET). Universidad Nacional del Litoral and Consejo Nacional de Investigaciones Científicas y Técnicas (CONICET). Esperanza, Santa Fe, Argentina. [mebaravalle@fcv.unl.edu.ar](mailto:mebaravalle@fcv.unl.edu.ar)

**Supporting Information**

**Figure S1:** Synthesis of PEGylated chrysin (PEG−C)

**Figure S2:** ^1^H NMR spectra for monotosylated PEG 400 g/mol in CDCl_3_

**Figure S3:** ^1^H NMR spectra for PEG−C in CDCl_3_

**Figure S4:** Cell viability. MTT assay in HepG2 cells treated with mPEG and PTX.

**Figure S1:** Synthesis of PEGylated chrysin (PEG−C)

Monotosylated PEG (400 g/mol) was synthesized according to a previously reported procedure (Pohlit et al. 2017). Briefly, PEGylated chrysin conjugates were prepared by a slightly modified literature method. Chrysin (1.1 mmol, 0.258 g) and K_2_CO_3_ (8 mmol, 1.023 g) were dissolved in 15 mL of dry DMF under a nitrogen atmosphere. Subsequently, the corresponding monotosylated PEG (1 mmol) was added under magnetic stirring, and the reaction mixture was heated at 70 °C for 24 h.

After cooling to room temperature, saturated NaHCO_3_ solution (50 mL) was added, and the mixture was extracted three times with dichloromethane. The combined organic phases were washed with brine, dried over anhydrous Na_2_SO_4_, and concentrated under reduced pressure. Residual DMF was removed by Kügelrohr distillation (70 °C, 70 mmHg). The resulting PEGylated chrysin product was dissolved in a minimal volume of dichloromethane (ca. 1 mL) and filtered through a nylon membrane filter (45 μm). A light brown oil was obtained.

Pohlit, H., Worm, M., Langhanki, J., Berger−Nicoletti, E., Opatz, T., & Frey, H. (2017). Silver Oxide Mediated Monotosylation of Poly(ethylene glycol) (PEG): Heterobifunctional PEG via Polymer Desymmetrization. *Macromolecules*, *50*(23), 9196−9206.<https://doi.org/10.1021/acs.macromol.7b01787>.

**Figure S2** ^1^H NMR spectra for monotosylated PEG 400 g/mol in CDCl_3_

^1^H NMR (400 MHz, CDCl_3_) monotosylated PEG (400 g/mol) *δ*: 2.44 (s, 3H); 3.55−3.90 (−polymer chain); 4.15 (t, *J* = 4.68 Hz, 2H); 7.35 (d, *J* =8.35 Hz, 2H) and 7.78 (d, *J* = 8.35 Hz, 2H) ppm correspond to the aromatic ring.

**Figure S3** ^1^H NMR spectra for PEG−C in DMSO−*d6*

^1^H NMR (CDCl_3_) **PEG−C** (*δ*): *δ*: 3.37 (s, 3H); 3.55−3.90 (−polymer chain); 4.24 (t, *J* = 4.15 Hz, 2H); 4.60 (s, 1H); 6.42 (s, 1H); 6.84 (s, 1H); 7.04 (s, 1H); 7.57−7.62 (m, 3H); 8.06−8.13 (m, 2H); 12.82 (bs, 1H).

**Figure S4:** Cell viability. MTT assay in HepG2 cells treated with mPEG and PTX.

Cell viability was assessed by the MTT assay in HepG2 cells treated with mPEG or PTX at 13, 33, 66, 133, and 265 µM. Each data point represents the percentage of viable cells expressed as mean ± SD. The dashed line denotes the 70% viability threshold.
